# Pulmonary and critical care fellowship applicants utilization of social media to evaluate programs

**DOI:** 10.1080/10872981.2019.1599277

**Published:** 2019-04-15

**Authors:** Timothy M. Dempsey, Kelly Pennington, Megan Dulohery-Scrodin, Kannan Ramar

To the Editor,

We are writing to share the results of a recent survey we completed regarding the use of social media by fellowship applicants to evaluate our Pulmonary and Critical Care Medicine program.

As social media use proliferates, medical education has tried to adapt, attempting to incorporate the different platforms into crucial areas such as education and recruitment [1]. Examples of this include using Twitter to promote national conferences and enhance local resident education [2,3]. While previous analysis has demonstrated that program directors may assess applicants based on their social media profiles, there has been no attempt to date to determine whether fellowship applicants evaluate programs based on the program’s social media presence [4].

To answer this question, we sent a survey to 51 individuals who interviewed at our institution during the 2018 application cycle. We also asked generally what influenced where applicants decided to apply, interview, and rank. A link to the survey was provided via several rounds of email. The survey was deemed exempt by the Institutional Review Board.

Of the 51 individuals surveyed, 25 completed the survey (49 percent response rate). Twenty two of the 25 respondents reported having at least one social media account with the most common use being Facebook (20/25) followed by Instagram (10/25) and Twitter (10/25). A large proportion of them (48 percent) described using social media to supplement their medical education. Only six out of 25 (24 percent) evaluated programs based on their social media page prior to deciding where to apply. The most common things applicants looked for when visiting a fellowship programs’ social media page was evidence of the success of current fellows, proof of the livability of the city the program was located in, and the quality of educational posts. When provided with an open-ended opportunity to answer what they wanted to see from programs’ social media accounts, the answers largely focused on the scholarship and social activities of current fellows. More broadly, when asked what factors influenced where they applied and interviewed, applicants felt clinical education (19/25), reputation of the program (16/25), and geography (16/25) were most important. When asked what factors influenced their rank list, applicants reported the perceived fit of the program (19/25), quality of clinical education (19/25), and geographic location (16/25) were most important.

While many applicants use social media to help augment their medical education, only a small percentage use it when deciding where to apply for fellowship. The more important things applicants use to decide where to interview, apply, and rank include the quality of clinical education, program reputation, and geographic location. The conduction of larger, nationwide surveys should be performed to better answer these important questions.
